# Alpha-Fetoprotein: From a Diagnostic Biomarker to a Key Role in Female Fertility

**Published:** 2007-02-07

**Authors:** Christelle De Mees, Julie Bakker, Josiane Szpirer, Claude Szpirer

**Affiliations:** 1 Université Libre de Bruxelles, Institut de Biologie et de Médecine Moléculaires, Rue Profs Jeener & Brachet, 12; B-6041 Gosselies (Charleroi), Belgium; 2 University of Liège, Center for Cellular & Molecular Neurobiology, Avenue de l’Hopital 1, B36; B-4000 Liège, Belgium

**Keywords:** Alpha-fetoprotein, Estrogens, Anovulation, Female brain differentiation

## Abstract

Alpha-fetoprotein (AFP) is a well-known diagnostic biomarker used in medicine to detect fetal developmental anomalies such as neural tube defects or Down’s syndrome, or to follow up the development of tumors such as hepatocellular carcinomas. However, and despite the fact that the protein was discovered almost half a century ago, little was known about its physiological function. The study of *Afp* knock-out mice uncovered a surprising function of AFP: it is essential for female fertility and for expression of normal female behaviors, and this action is mediated through its estrogen binding capacity. AFP sequestrates estrogens and by so doing protects the female developing brain from deleterious (defeminizing/masculinizing) effects of these hormones.

Alpha-fetoprotein (AFP), discovered about half a century ago (Bergstrand and Czar, 1956; Abelev et al. 1963), is the major serum fetal protein in mammals. AFP is actively produced and secreted during the fetal life by the liver hepatocytes, the visceral endoderm of the yolk sac and, to a lesser extent, by the intestine and the kidneys (Sell and Becker, 1978; Andrews et al. 1982; Belayew and Tilghman, 1982). The concentration of this protein in the fetal serum reaches the order of several mg/ml, and its synthesis decreases dramatically in the first weeks after birth to reach only trace amounts in adulthood (Sell and Becker, 1978; Belayew and Tilghman, 1982). It is then essentially produced by the liver.

AFP produced by the embryo is secreted in the amniotic fluid and is also able to cross the placental barrier to reach the maternal blood circulation, where its titer is used as a diagnostic marker to reveal developmental anomalies of the fetus (Haddow et al. 1979; Brownbill et al. 1995; Newby et al. 2005). Abnormally high levels of AFP in the maternal serum indicates elevated risk for neural tube defects of the fetus such as spina bifida or anencephaly (Leighton et al. 1975), whereas abnormally low levels indicates elevated risk for a Down’s syndrome (Cuckle et al. 1984). Measurements of the AFP levels in the maternal serum are undertaken at 14–22 weeks of each pregnancy and are part, along with unconjugated estradiol, human chorionic gonadotropin and inhibin A, of the quadruple test for antenatal Down’s syndrome screening (Wald et al. 2003). Abnormal AFP levels can also be indicative of other fetal pathologies (for review see Mizejewski, 2004).

Synthesis of AFP is dramatically reduced in adulthood but can resume in case of liver pathologies (cirrhosis, hepatitis…) or of tumors such as hepatocellular carcinoma, germ cell tumors (embryonic carcinoma and teratocarcinoma) and some pancreatic and renal tumors (Masopust et al. 1968; Chiu et al. 1983; Abelev and Eraiser, 1999; Labdenne and Heikinheimo, 2002; Yuen and Lai, 2005; Ishigami et al. 2006). Little is known about the exact mechanisms that control the AFP gene expression or silencing (for reviews see Lazarevich, 2000; Spear, 1999). The regulatory region of the AFP gene contains distal enhancers, a promoter element, and silencer elements (Godbout et al. 1986; Watanabe et al. 1988; Poliard et al. 1990; Vacher and Tilghman, 1990; Henriette et al. 1997). The promoter element is regulated by numerous transcriptional factors such as HNF1 and HNF3 (Feuerman et al. 1989; Zhang et al. 1990; Crowe et al. 1999), Nkx2.8 (Apergis et al. 1998), FTF (Galerneau et al. 1996), NF1 (Bernier et al. 1993), Zhx2 (Perincheri et al. 2005), FOXA (Huang et al. 2002), the thyroid hormone receptor (Van Reeth et al. 2002) and Ku (Lienard et al. 2006). The thyroid hormone T3 probably contributes to the post-natal shut off of the gene (Van Reeth et al. 2002).

Until recently, the precise function of AFP was unknown. In order to identify this function, *Afp* knock-out mice were generated (Gabant et al. 2002) by replacing an *Afp* genomic fragment extending from exon 1, to intron 3 (*Afp^tm1Ibmm^* allele), or extending from exon 2 to intron 3 (*Afp^tm2Ibmm^* allele), by a IRES-*LacZ-neo* selection cassette. Both invalidations gave rise to viable homozygous animals. These AFP KO mice are apparently normal, but females are sterile, while males are fertile.

AFP KO female mice suffer from anovulation. Reciprocal ovary transplantation experiments demonstrated that AFP KO ovaries are functional: AFP KO ovaries transplanted in normal mice were able to ovulate and the transplanted females generated pups from the mutated parental oocytes. AFP KO ovaries contain follicles at different stages of maturation, including the last Grafiaan follicle stage. However no corpora lutea, indicative of ovulation, could be detected, which is in accordance with the smooth exterior aspect of the ovaries and the abnormally low levels of progesterone in the serum. Ovulation in AFP KO mice can be induced by injection of gonadotropins. In that case, ova are released, fertilized, but the blastocysts are unable to implant in the uterine horns which are non-receptive because of overstimulation by estrogens, as a result of the absence of corpora lutea.

Since the AFP KO female mice defect does not lie within the ovaries, it must implicate the HPG axis (hypothalamic-pituitary-gonadal axis), which provides the adequate hormonal environment necessary for ovulation. In the proestrus phase of the sexual cycle, the HPG axis responds to a stimulatory signal of the estrogens by the secretion of the GnRH decapeptide which binds to its receptor in the pituitary and triggers the release of the LH and FSH hormones responsible, in fine, for ovulation. In order to define the defect of the HPG axis in the AFP KO females, we used the micro-array technique to compare the gene expression profile of these mice with that of their wild type littermates. We found that in the pituitary, several genes previously implicated in female fertility are down-regulated in the AFP KO female mice (De Mees et al. 2006). These genes are *Egr1*, *Cish2*, *Ptprf*, *Psa*, and *Tkt*. Furthermore, we also found that genes participating in the GnRH pathway are downregulated in the AFP KO female mice. These genes are the GnRH receptor gene, and several genes activated by the GnRH receptor (*cFos*, *Egr2*, *Tgfb1i*4, *Ptp4a1*). In the hypothalamus, the gene encoding the hypothalamic GnRH decapeptide is itself down-regulated. In the context of an anovulation phenotype, dysfunction of the GnRH pathway seems extremely relevant.

In addition to being sterile, female AFP KO mice are defeminized (they show a diminution of female behavior) and masculinized (they exhibit some male characteristics): in the presence of a sexually active male, they do not exhibit the female typical behavior of lordosis (posture with raised head and rump, and deflected tail, to facilitate copulation) and they show a male pattern of distribution of tyrosine hydroxylase expressing neurons in sexually dimorphic areas of the hypothalamus (Bakker et al. 2006).

## What Can be the Link Between AFP and Female Fertility?

The answer lies in the capacity of AFP to inhibit estrogen responsiveness. AFP is able, at least in rodents, to bind estrogens, but not androgens, at its C-terminal extremity, with a *K_d_* of 10^−9^ M^−1^, indicating that it can act as an estrogen carrier in the blood (Uriel et al. 1976; Savu et al. 1981; Nishi et al. 1991). Estrogens are known to exert deleterious, masculinizating effects on the female developing brain in the perinatal period. Perinatal exposure to estrogens in rodent females results in anovulatory sterility in adulthood, associated with altered gonadotropin production and absence of female typical behaviors (Gorski, 1963; Whaler and Nadler, 1963). Based on the observation, it is classically assumed that the function of AFP is to shield the female brain from estrogens, by sequestrating them in the fetal serum (Mc Ewen et al. 1975). As AFP is unable to bind androgens, testosterone produced by the male embryo’s testes is free to reach the developing brain where it can locally be converted to estrogens by an enzyme called aromatase, and according to this classic hypothesis, estrogens could thus exert their masculinizating effects. An alternative hypothesis, based on the fact that AFP is found inside neurons without being produced locally, is that AFP has more than a neuroprotective role and specifically delivers estrogens to targeted brain cells in order to ensure correct female differentiation (Dohler et al. 1984; Toran-Allerand, 1984). The AFP KO mice allowed us to test these hypotheses.

We reasoned that if AFP was essentially a passive estrogen carrier, the fertility of the AFP KO female mice should be restored provided their embryonic development took place in an environment strongly reduced in oestrogens; alternatively, if AFP was an active estrogen carrier, then the lack of both estrogens and AFP should be as deleterious as the lack of AFP alone. Gestation is compatible with very low level of estrogens, which can be reached by treating gestating females with an aromatase inhibitor. We found that the fertility of the so-treated AFP KO female mice was restored. No treatment was needed in adulthood to sustain this fertility, thereby proving that the cause of anovulation in AFP KO mice is indeed estrogen overexposure. Furthermore, normal gene expression in the HPG, normal lordosis behavior, and normal distribution of the tyrosine hydroxylase expressing neurons were also regained in the treated females (Bakker et al. 2006; De Mees et al. 2006). These results demonstrate that AFP has merely a passive, neuroprotective role, and protects the female developing brain from estrogen deleterious effects.

However, these findings do not explain why AFP is found inside neurons without being locally produced. AFP could have other neurological functions, independent from fertility control. Our results do not exclude the possibility of an active post natal role of estrogen in female differentiation. Indeed, females mice ovariectomized at birth display less feminine behaviors than intact females in adulthood (Gerall et al. 1973). This hypothesis is supported by the finding that aromatase knockout females are sterile and remain so even after adult estradiol treatment (Toda et al. 2001). Lastly, very low quantities of estrogens could indeed be needed for correct female brain sexual differentiation, but in that case, they would be transported by another carrier than AFP.

Finally, it should be pointed out that the anti-estrogenic effects of AFP could go beyond its estrogen binding and sequestrating properties, as deduced from experiments which have shown an AFP induced hypo responsiveness to estrogens in the presence of molar excess of estrogens over AFP (Mizejewski et al. 1983).

Translation of the observed results to human still needs to be tested. There are diverging views in the literature as to whether human AFP can or cannot bind estrogens. In either case, it appears that human AFP-derived peptides are able to display some anti-estrogenic activity (Vakharia and Mizejewski, 2000; Bennett et al. 2002; Mizejewski et al. 2004). AFP-derived peptides are under investigation as chemopreventive agents for estrogen-dependent breast cancers and other tumors (Bennett et al. 2006, Mizejewski et al. 2006). Androgens could also, in the human, play a more important role than estrogens in sexual brain differentiation. In that case the sex hormone binding globulin, able to bind both estrogens and androgens, could then play a significant role.

In conclusion, our work demonstrates that the function of AFP extends well beyond its traditional marker role for developmental anomalies of the fetus or liver tumors. AFP plays a crucial role (at least in rodents) in the control of female fertility through its anti-estrogenic action.

## References

[b1-bmi-2006-085] Abelev GI Eraiser TL 1999 Cellular aspects of alpha-fetoprotein reexpression in tumors Semin Cancer Biol 9 95 107 10202131 10.1006/scbi.1998.0084

[b2-bmi-2006-085] Abelev GI Perova SD Khramkova NI Postnikova ZA Irlin IS 1963 Production of embryonal α-globulin by transplantable mouse hepatomas Transplantation 1 174 180 14010646 10.1097/00007890-196301020-00004

[b3-bmi-2006-085] Andrews GK Dziadek M Tamaoki T 1982 Expression and methylation of the mouse alpha-fetoprotein gene in embryonic, adult, and neoplastic tissues J Biol Chem 257 5148 5153 6175646

[b4-bmi-2006-085] Apergis GA Crawford N Ghosh D 1998 A novel nk-2-related transcription factor associated with human fetal liver and he-patocellular carcinoma J Biol Chem 273 2917 2925 9446603 10.1074/jbc.273.5.2917

[b5-bmi-2006-085] Bakker J De Mees C Douhard Q 2006 Alpha-fetoprotein protects the developing female mouse brain from masculinization and defeminization by estrogens Nat Neurosci 9 220 226 16388309 10.1038/nn1624

[b6-bmi-2006-085] Belayew A Tilghman SM 1982 Genetic analysis of alpha-fetoprotein synthesis in mice Mol Cell Biol 2 1427 1435 6186903 10.1128/mcb.2.11.1427-1435.1982 PMC369947

[b7-bmi-2006-085] Bennett JA Mesfin FB Andersen TT 2002 A peptide derived from alpha-fetoprotein prevents the growth of estrogen-dependent human breast cancers sensitive and resistant to tamoxifen Proc Natl Acad Sci USA 99 2211 5 11830647 10.1073/pnas.251667098 PMC122344

[b8-bmi-2006-085] Bennett JA Defreest L Anaka I 2006 AFPep: an anti-breast cancer peptide that is orally active Breast Cancer Res Treat10.1007/s10549-005-9140-5 16538538

[b9-bmi-2006-085] Bergstrand CG Czar B 1956 Demonstration of a new protein fraction in serum from the human fetus Scand J Clin Lab Invest 8 174 179 13351554 10.3109/00365515609049266

[b10-bmi-2006-085] Bernier D Thomassin H Allard D 1993 Functional analysis of developmentally regulated chromatin-hypersensitive domains carrying the alpha 1-fetoprotein gene promoter and the albumin/alpha 1-fetoprotein intergenic enhancer Mol Cell Biol 13 1619 1633 7680097 10.1128/mcb.13.3.1619-1633.1993 PMC359474

[b11-bmi-2006-085] Brownbill P Edwards D Jones C 1995 Mechanisms of alphafetoprotein transfer in the perfused human placental cotyledon from uncomplicated pregnancy J Clin Invest 96 2220 2226 7593608 10.1172/JCI118277 PMC185872

[b12-bmi-2006-085] Chiu JF Huang DP Burkhardt AL 1983 The alteration of gene expression in rat liver during chemical carcinogenesis Arch Biochem Biophys 222 310 320 6838226 10.1016/0003-9861(83)90528-3

[b13-bmi-2006-085] Crowe AJ Sang L Li KK Lee KC Spear BT Barton MC 1999 Hepatocyte nuclear factor 3 relieves chromatin-mediated repression of the α-fetoprotein gene J Biol Chem 274 25113 25120 10455192 10.1074/jbc.274.35.25113

[b14-bmi-2006-085] Cuckle HS Wald NJ Lindenbaum RH 1984 Maternal serum alpha-fetoprotein measurement: a screening test for Down syndrome Lancet 1 926 929 6201687 10.1016/s0140-6736(84)92389-4

[b15-bmi-2006-085] De Mees C Laes JF Bakker J 2006 Alpha-fetoprotein controls female fertility and prenatal development of the gonadotropin-releasing hormone pathway through an antiestrogenic action Mol Cell Biol 26 2012 2018 16479017 10.1128/MCB.26.5.2012-2018.2006 PMC1430253

[b16-bmi-2006-085] Döhler KD Hancke JL Srivastava SS 1984 Participation of estrogens in female sexual differentiation of the brain; neuroanatomical, neuroendocrine and behavioral evidence Prog Brain Res 61 99 117 6084848 10.1016/S0079-6123(08)64430-1

[b17-bmi-2006-085] Feuerman MH Godbout R Ingram RS 1989 Tissue-specific transcription of the mouse alpha-fetoprotein gene promoter is dependent on HNF-1 Mol Cell Biol 9 4204 4212 2479822 10.1128/mcb.9.10.4204-4212.1989 PMC362499

[b18-bmi-2006-085] Gabant P Forrester L Nichols J 2002 Alpha-fetoprotein, the major fetal serum protein, is not essential for embryonic development but is required for female fertility Proc Natl Acad Sci USA 99 12865 12870 12297623 10.1073/pnas.202215399 PMC130551

[b19-bmi-2006-085] Galarneau L Pare JF Allard D 1996 The alpha1-fetoprotein locus is activated by a nuclear receptor of the Drosophila FTZ-F1 family Mol Cell Biol 16 3853 3865 8668203 10.1128/mcb.16.7.3853 PMC231382

[b20-bmi-2006-085] Gerall AA Dunlap JL Hendricks SE 1973 Effect of ovarian secretions on female behavioral potentiality in the rat J Comp Physiol Psychol 1973 82 449 65 4735922 10.1037/h0034113

[b21-bmi-2006-085] Godbout R Ingram R Tilghman SM 1986 Multiple regulatory elements in the intergenic region between the alpha-fetoprotein and albumin genes Mol Cell Biol 6 477 487 2431269 10.1128/mcb.6.2.477-487.1986 PMC367536

[b22-bmi-2006-085] Gorski RA 1963 Modification of ovulatory mechanisms by postnatal administration of estrogen to the rat Am J Physiol 205 842 844 5896305 10.1152/ajplegacy.1963.205.5.842

[b23-bmi-2006-085] Haddow JE Macri JN Munson M 1979 The amnion regulates movement of fetally derived alpha-fetoprotein into maternal blood J Lab Clin Med 94 344 347 88493

[b24-bmi-2006-085] Henriette MF Gabant P Drèze P Szpirer C Szpirer J 1997 Negative regulation of the α-foetoprotein gene in fibroblasts: identification and characterization of *cis* and *trans* elements Folia Biologica 43 5 13 9158943

[b25-bmi-2006-085] Huang MC Li KK Spear BT 2002 The mouse alpha-fetoprotein promoter is repressed in HepG2 hepatoma cells by hepatocyte nuclear factor-3 (FOXA) DNA Cell Biol 21 561 569 12215259 10.1089/104454902320308933 PMC1563500

[b26-bmi-2006-085] Ishigami S Natsugoe S Nakashima H 2006 Biological aggressiveness of alpha-fetoprotein (AFP)-positive gastric cancer Hepatogastroenterology 53 338 341 16795967

[b27-bmi-2006-085] Labdenne P Heikinheimo M 2002 Clinical use of tumor markers in childhood malignancies Ann Med 34 316 323 12452476 10.1080/078538902320772070

[b28-bmi-2006-085] Lazarevich NL 2000 Molecular mechanisms of alpha-fetoprotein gene expression Biochemistry 65 117 133 10702646

[b29-bmi-2006-085] Leighton PC Kitau MJ Chard T 1975 Levels of alpha-feto-protein in maternal blood as a screening test for fetal neural-tube defect Lancet 2 1012 1015 53498 10.1016/s0140-6736(75)90295-0

[b30-bmi-2006-085] Lienard P De Mees C Dreze PL 2006 Regulation of the alpha-fetoprotein promoter: Ku binding and DNA spatial conformation Biochimie10.1016/j.biochi.2006.05.006 16765502

[b31-bmi-2006-085] Masopust J Kithier K Radl J 1968 Occurrence of fetoprotein in patients with neoplasms and non-neoplastic diseases Int J Cancer 3 364 373 4176107 10.1002/ijc.2910030306

[b32-bmi-2006-085] McEwen BS Plapinger L Chaptal C 1975 Role of fetoneo-natal estrogen binding proteins in the associations of estrogen with neonatal brain cell nuclear receptors Brain Res 96 400 406 169964 10.1016/0006-8993(75)90755-6

[b33-bmi-2006-085] Mizejewski GJ Vonnegut M Jacobson HI 1983 Estradiol-activated alpha-fetoprotein suppresses the uterotropic response to estrogens Proc Natl Acad Sci USA 80 2733 2737 6189129 10.1073/pnas.80.9.2733 PMC393902

[b34-bmi-2006-085] Mizejewski GJ 2004 Biological roles of alpha-fetoprotein during pregnancy and perinatal development Exp Biol Med 229 439 463 10.1177/153537020422900602 15169963

[b35-bmi-2006-085] Mizejewski GJ Smith G Butterstein G 2004 Review and proposed action of alpha-fetoprotein growth inhibitory peptides as estrogen and cytoskeleton-associated factors Cell Biol Int 28 913 933 15566961 10.1016/j.cellbi.2004.09.005

[b36-bmi-2006-085] Mizejewski GJ Muehlemann M Dauphinee M 2006 Update of alpha fetoprotein growth-inhibitory peptides as biotherapeutic agents for tumor growth and metastasis Chemotherapy 52 83 90 16498241 10.1159/000091728

[b37-bmi-2006-085] Newby D Dalgliesh G Lyall F 2005 Alphafetoprotein and alphafetoprotein receptor expression in the normal human placenta at term Placenta 26 190 200 15708120 10.1016/j.placenta.2004.06.005

[b38-bmi-2006-085] Nishi S Matsue H Yoshida H 1991 Localization of the estrogen-binding site of alpha-fetoprotein in the chimeric human-rat proteins Proc Natl Acad Sci USA 88 3102 3105 1707533 10.1073/pnas.88.8.3102 PMC51393

[b39-bmi-2006-085] Perincheri S Dingle RW Peterson ML 2005 Hereditary persistence of alpha-fetoprotein and H19 expression in liver of BALB/cJ mice is due to a retrovirus insertion in the Zhx2 gene Proc Natl Acad Sci USA 102 396 401 15626755 10.1073/pnas.0408555102 PMC544306

[b40-bmi-2006-085] Poliard A Bakkali L Poiret M Foiret D Danan JL 1990 Regulation of the rat α-fetoprotein gene expression in liver J Biol Chem 265 2137 2141 1688847

[b41-bmi-2006-085] Savu L Benassayag C Vallette G 1981 Mouse alpha 1-feto-protein and albumin. A comparison of their binding properties with estrogen and fatty acid ligands J Biol Chem 256 9414 9418 6169710

[b42-bmi-2006-085] Sell S Becker FF 1978 Alpha-Fetoprotein J Natl Cancer Inst 60 19 26 75267 10.1093/jnci/60.1.19

[b43-bmi-2006-085] Spear BT 1999 Alpha-fetoprotein gene regulation: lessons from transgenic mice Semin Cancer Biol 9 109 16 10202132 10.1006/scbi.1998.0087

[b44-bmi-2006-085] Toda K Takeda K Okada T 2001 Targeted disruption of the aromatase P450 gene (Cyp19) in mice and their ovarian and uterine responses to 17beta-oestradiol J Endocrinol 170 99 111 11431142 10.1677/joe.0.1700099

[b45-bmi-2006-085] Toran-Allerand CD 1984 On the genesis of sexual differentiation of the general nervous system: morphogenetic consequences of steroidal exposure and possible role of alpha-fetoprotein Prog Brain Res 61 63 98 6084847 10.1016/s0079-6123(08)64429-5

[b46-bmi-2006-085] Uriel J Bouillon D Aussel C Dupiers M 1976 Alpha-fetoprotein: the major high-affinityestrogen binder in rat uterine cytosols Proc Natl Acad Sci USA 73 1452 1456 58416 10.1073/pnas.73.5.1452 PMC430314

[b47-bmi-2006-085] Vacher J Tilghman SM 1990 Dominant negative regulation of the mouse alpha-fetoprotein gene in adult liver Science 250 1732 1735 1702902 10.1126/science.1702902

[b48-bmi-2006-085] Vakharia D Mizejewski GJ 2000 Human alpha-fetoprotein peptides bind estrogen receptor and estradiol, and suppress breast cancer Breast Cancer Res Treat 63 41 52 11079158 10.1023/a:1006484223325

[b49-bmi-2006-085] Van Reeth T Gabant P Szpirer C Szpirer J 2002 Stimulation of the alpha-fetoprotein promoter by unliganted thyroid hormone receptor in association with protein deacetylation Mol Cell Endocrin 188 99 109 10.1016/s0303-7207(01)00739-0 11911950

[b50-bmi-2006-085] Whalen RE Nadler RD 1963 Suppression of the development of female mating behavior by estrogen administered in infancy Science 141 273 274 14000192 10.1126/science.141.3577.273

[b51-bmi-2006-085] Wald NJ Huttly WJ Hackshaw AK 2003 Antenatal screening for Down’s syndrome with the quadruple test Lancet 361 835 836 12642052 10.1016/S0140-6736(03)12680-3

[b52-bmi-2006-085] Watanabe K Saito A Tamaoki T 1988 Cell-specific enhancer activity in a far upstream region of the human α-fetoprotein gene J Biol Chem 262 4812 4818 2435718

[b53-bmi-2006-085] Yuen MF Lai CL 2005 Serological markers of liver cancer Best Pract Res Clin Gastroenterol 19 91 99 15757806 10.1016/j.bpg.2004.10.003

[b54-bmi-2006-085] Zhang DE Hoyt PR Papaconstantinou J 1990 Localization of DNA protein-binding sites in the proximal and distal promoter regions of the mouse alpha-fetoprotein gene J Biol Chem 265 3382 3391 1689301

